# Loss of Functionally Redundant p38 Isoforms in T Cells Enhances Regulatory T Cell Induction*

**DOI:** 10.1074/jbc.M116.764548

**Published:** 2016-12-23

**Authors:** Morisada Hayakawa, Hiroko Hayakawa, Tsvetana Petrova, Patcharee Ritprajak, Ruhcha V. Sutavani, Guillermina Yanek Jiménez-Andrade, Yasuyo Sano, Min-Kyung Choo, John Seavitt, Ram K. C. Venigalla, Kinya Otsu, Katia Georgopoulos, J. Simon C. Arthur, Jin Mo Park

**Affiliations:** From the ‡ Cutaneous Biology Research Center, Massachusetts General Hospital and Harvard Medical School, Charlestown, Massachusetts 02129,; the § Department of Biochemistry, Jichi Medical University, Shimotsuke, Tochigi 329-0498, Japan,; the ¶ Division of Cell Signalling and Immunology, School of Life Sciences, Wellcome Trust Building, and; ** MRC Protein Phosphorylation Unit, School of Life Sciences, Sir James Black Centre, University of Dundee, Dundee DD1 5EH, United Kingdom,; the ‖ Department of Microbiology and Immunology and Research Unit of Oral Microbiology, Faculty of Dentistry, Chulalongkorn University, Bangkok 10330, Thailand, and; the ‡‡ Cardiovascular Division, King's College London, London SE5 9NU, United Kingdom

**Keywords:** immunology, mouse, p38, signal transduction, T-cell

## Abstract

The evolutionarily conserved protein kinase p38 mediates innate resistance to environmental stress and microbial infection. Four p38 isoforms exist in mammals and may have been co-opted for new roles in adaptive immunity. Murine T cells deficient in p38α, the ubiquitously expressed p38 isoform, showed no readily apparent cell-autonomous defects while expressing elevated amounts of another isoform, p38β. Mice with T cells simultaneously lacking p38α and p38β displayed lymphoid atrophy and elevated Foxp3^+^ regulatory T cell frequencies. Double deficiency of p38α and p38β in naïve CD4^+^ T cells resulted in an attenuation of MAPK-activated protein kinase (MK)-dependent mTOR signaling after T cell receptor engagement, and enhanced their differentiation into regulatory T cells under appropriate inducing conditions. Pharmacological inhibition of the p38-MK-mTOR signaling module produced similar effects, revealing potential for therapeutic applications.

## Introduction

The T cell receptor (TCR)^3^ and receptors for costimulatory molecules and cytokines deliver key signals for the development and function of T cells. Upon ligand binding, these receptors trigger intracellular signaling cascades that are integrated to elicit changes in gene expression and cellular phenotype. In peripheral naïve T cells, for example, antigen encounter and costimulation in conjunction with exposure to specific cytokines lead to the induction of a gene transcription program that specifies differentiation into a functionally distinct subset of effector, memory, or regulatory cells. The magnitude as well as the nature of receptor-initiated signals contributes to determining the fate and functional capabilities of activated T cells. Modulating intracellular signaling in T cells by pharmacological means can dampen, augment, or re-route immune responses, an approach that may be harnessed to suppress immune-mediated disorders and enhance antitumor immunity.

Homologues of p38, a MAPK family member, are found in organisms ranging from yeast to humans. In single-celled life, p38 is activated upon stress and damage, and signals to evoke cell-autonomous mechanisms for adaptation, repair, and survival (1, 2). The role of p38 signaling in multicellular organisms extends to antimicrobial defense, with p38-dependent phosphorylation events at work downstream of receptors for immune signals (3–5). In most physiological contexts, p38 activation in mammalian cells occurs through a “classical” MAPK cascade, in which p38 is phosphorylated by the MAPK kinases MKK3 and MKK6, which themselves are activated by stimulus-specific MAPK kinase kinase-mediated phosphorylation (6). Four mammalian p38 isoforms (p38α, p38β, p38γ, and p38δ) have evolved to assume specialized but partly redundant physiological roles. Of particular importance to immune function is p38α, the isoform most widely expressed in tissues (7, 8) and involved in cell responses to cytokines and microbial products (9–11). Moreover, p38α was originally identified as the molecular target of a class of small-molecule anti-inflammatory compounds (12), and since this initial finding p38α has been implicated in signaling processes associated with a host of diseases of inflammatory etiology (6). Cell type-specific loss of p38α signaling in mice, with p38α gene ablation restricted to epithelial cells, myeloid cells, or dendritic cells, was found to prevent or ameliorate inflammatory responses and pathology in ultraviolet radiation-induced dermatitis (13, 14), chemically induced colitis (15), contact hypersensitivity reactions (16), and experimental autoimmune encephalomyelitis (17).

The activation of p38 in T cells occurs not only through the classical MKK3/6-mediated process (18–20) but also via an alternative pathway involving p38 phosphorylation by the TCR-proximal tyrosine kinase ZAP70 (21). ZAP70-activated p38α and p38β serve some redundant functions in T cells (22), and their activating phosphorylation sites and substrate specificities differ from those of MKK3/6-activated counterparts (23). Several studies examined the *in vivo* effects of impaired p38 function in T cells by using pharmacological p38 inhibitors (24, 25), dominant-negative p38α and MKK transgenes (19, 24–27), p38α- and MKK-null alleles (18, 28–30), and p38 gene knock-in alleles selectively precluding alternative activation (22, 31, 32). The findings from these approaches suggested a role for T cell p38 signaling in thymocyte development, TCR-induced proliferation and apoptosis, IFN-γ, IL-2, and IL-17A production, and autoimmune diseases such as collagen-induced arthritis and experimental autoimmune encephalomyelitis. Other studies that examined mice with T cells lacking p38α alone or both p38α and p38β, however, did not observe substantial effects on IFN-γ and IL-17A production or experimental autoimmune encephalomyelitis (17). The role of p38 signaling in T cells, therefore, remains debatable, its potential as a target for anti-inflammatory therapy yet to be definitely appraised.

In this study, we find as-yet-unreported effects of ablating p38α and p38β in T cells: mice with T cells simultaneously deficient in the two p38 isoforms exhibit enhanced regulatory T (Treg) cell induction and attenuated allergic inflammation when challenged with epicutaneous antigen. *In vitro* differentiation experiments confirm the role of p38 signaling in limiting Treg cell induction, and identify how p38α and p38β cooperate to perform this role. Our findings suggest inhibition of p38 signaling as a novel means to promote Treg cell generation and treat immune-mediated diseases.

## Results

### 

#### 

##### Development and Maintenance of T Cells Lacking p38α and p38β

**FIGURE 1.**
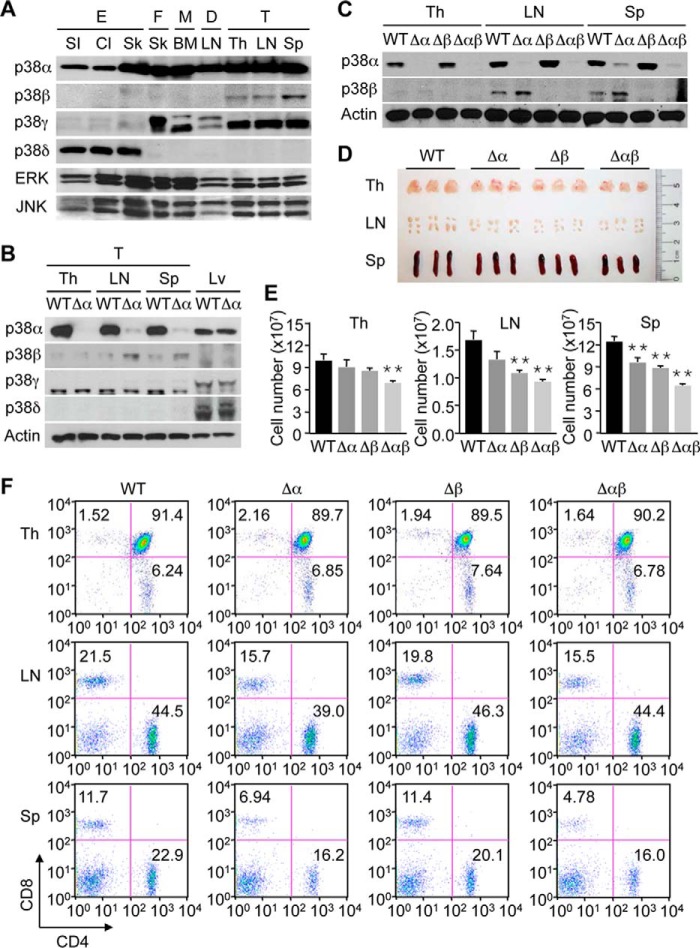
**Mice with p38α and p38β deficiency in T cells display lymphoid atrophy and a decreased proportion of lymph node and splenic CD8^+^ T cells.** *A*, whole cell lysates from epithelial cells (*E*), fibroblasts (*F*), macrophages (*M*), dendritic cells (*D*), and T cells (*T*) were analyzed by immunoblotting. The cells were isolated from the small intestine (*SI*), colon (*Cl*), skin (*Sk*), lymph node (*LN*), thymus (*Th*), and spleen (*Sp*) of the mouse, or derived by *in vitro* differentiation of progenitors in the mouse bone marrow (*BM*). *B* and *C*, whole cell lysates from T cells (*T*) of the indicated mouse tissues and from liver tissue (*Lv*) were analyzed by immunoblotting. *D* and *E*, indicated mouse tissues (*n* = 3, each group) were photographed (*D*), and their cell numbers were determined (*E*). Cell numbers shown as mean ± S.D. (*n* = 3, each group). **, *p* < 0.01. *F*, percentage of CD4^+^ and CD8^+^ cells in the indicated mouse tissues was determined by flow cytometry. Data are representative of two (*A–C*) or three (*D* and *E*) experiments and of six animals (*F*) with similar results.

We previously reported that mice with T cell-specific ablation of p38α (*Mapk14^fl/fl^-LckCre*; Δα) showed moderate lymphoid atrophy, and had decreased proportions of CD8^+^ subsets among lymph node and splenic T cells (16). Combined, these changes translated to a decline in the absolute number of CD8^+^ T cells available for antigen-specific immune responses such as contact hypersensitivity reactions. Nevertheless, T cells from these mice, taken individually, remained functionally competent throughout the immune response: in hapten-exposed animals, p38α-deficient T cells were efficiently primed to become cytokine-producing effector cells, and formed a memory population that could transfer hapten sensitivity to naïve mice (16). This may be due either to a lack of critical cell-autonomous functions of p38α in T cells during immune responses or to functional compensation by other p38 isoforms. Such functional redundancy has been documented for p38α and p38β in various cell types including T cells (22, 33). Of note, p38β expression in mice was prominent in T cells compared with other cell types (Fig. 1*A*), and further elevated in lymph node and splenic T cells with p38α ablation (Fig. 1*B*).

To examine whether p38α and p38β exert redundant functions in T cells, we crossed Δα mice with p38β-null mice (*Mapk11*^−/−^; Δβ) to generate mice with T cells lacking both p38α and p38β (*Mapk14^fl/fl^-LckCre-Mapk11*^−/−^; Δαβ). T cells from Δαβ mice did not express detectable amounts of both p38 isoforms (Fig. 1*C*). The size and cellularity of the thymus, lymph nodes, and spleen of Δαβ mice decreased compared with those of wild-type (WT) mice (Fig. 1, *D* and *E*). The lymphoid tissues of Δα and Δβ mice tended to be atrophic too, but to lesser extents than Δαβ counterparts. Single and double deficiency of p38α and p38β in T cells did not prevent the thymic development of CD4^+^ and CD8^+^ T cells or their homing to and maintenance in the lymph nodes and spleen (Fig. 1*F*). In keeping with our previous report (16), the proportions of lymph node and splenic CD8^+^ T cells were lower in Δα mice than in WT mice, but not further reduced by the additional loss of p38β in Δαβ mice (Fig. 1*F*).

##### Signaling by p38α and p38β in T Cells

**FIGURE 2.**
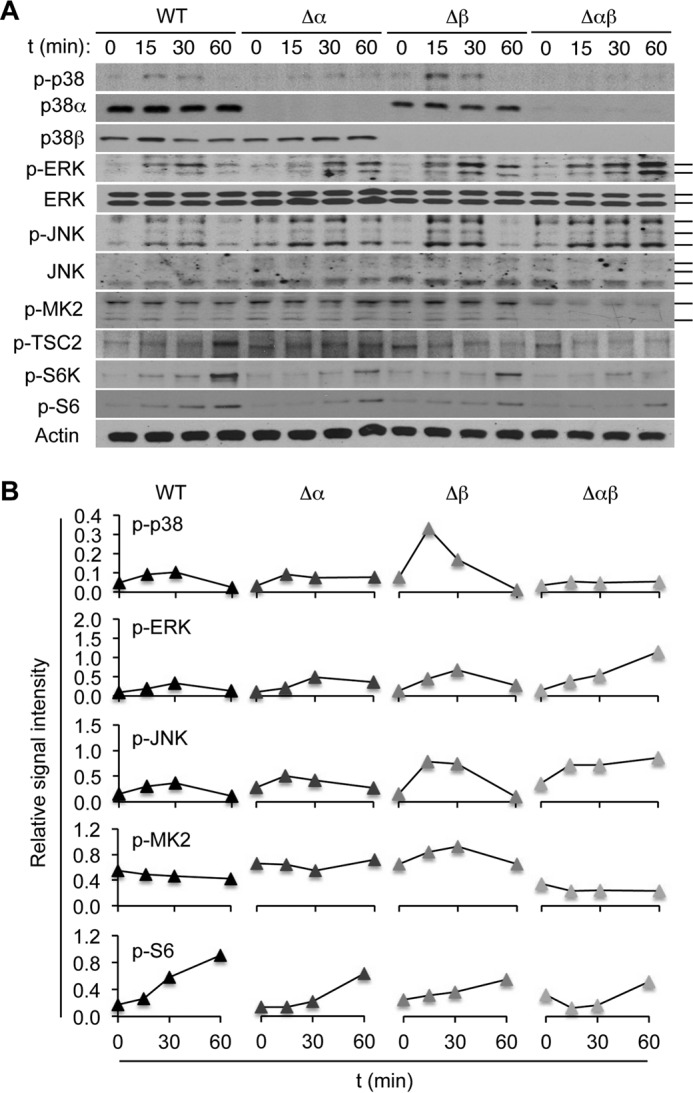
**p38α and p38β cooperate to shape TCR-induced intracellular signaling in CD4^+^ T cells.** *A* and *B*, naïve CD4^+^ T cells from the indicated mice were left unstimulated or stimulated with anti-CD3 and anti-CD28. Whole cell lysates were prepared after the indicated durations of stimulation and analyzed by immunoblotting (*A*). *Solid bars* on the right indicate bands corresponding to multiple protein isoforms detected by the antibodies. *p-*, phosphorylated. Immunoblot signals were quantified by densitometry, and the relative signal intensities (the indicated proteins relative to actin) are shown (*B*). Data are representative of three experiments with similar results (*A* and *B*).

We examined the effects of p38 deficiency on TCR-induced intracellular signaling in naïve CD4^+^ T cells. Compared with T cells lacking only either isoform, T cells from Δαβ mice exhibited a more complete abrogation of p38 phosphorylation after treatment with TCR agonists, anti-CD3 and anti-CD28 antibodies (Fig. 2, *A* and *B*). Loss of p38 has been shown to potentiate signaling by other MAPK family members, ERK and JNK, in a multitude of cell types (34). Correspondingly, Δαβ T cells showed increased phosphorylation, and thus activation, of ERK and JNK upon TCR stimulation (Fig. 2*A*). We observed that MAPK-activated protein kinase (MK) 2, a protein kinase immediately downstream of p38, was constitutively phosphorylated in cultured T cells independently of TCR stimulation. Nevertheless, MK2 phosphorylation was abolished in Δαβ T cells, but not Δα and Δβ T cells (Fig. 2, *A* and *B*), suggesting that p38 was indispensable for steady-state phosphorylation of MK2.

**FIGURE 3.**
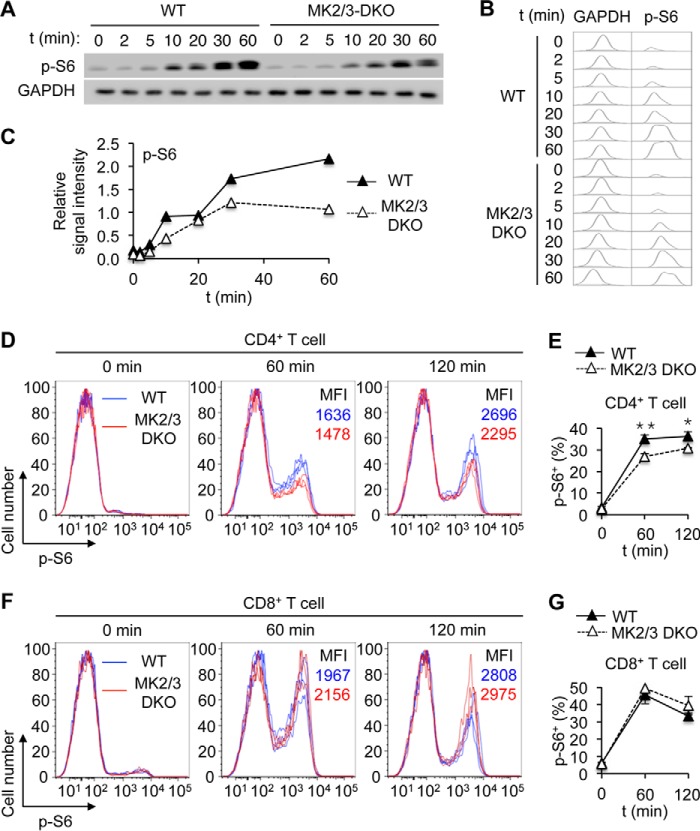
**MK2/3 signaling promotes TCR-induced mTOR pathway activation in CD4^+^ T cells.** *A–C*, naïve CD4^+^ T cells from the indicated mice were left unstimulated or stimulated with anti-CD3 and anti-CD28. Whole cell lysates were prepared after the indicated durations of stimulation and analyzed by immunoblotting (*A*). *p-*, phosphorylated. Immunoblots were scanned and shown in histograms (*B*). Immunoblot signals were quantified by densitometry, and the relative signal intensities (p-S6 relative to GAPDH) are shown (*C*). *D–G*, splenocytes from the indicated mice were treated with anti-CD3 for the indicated durations. Relative p-S6 amounts in CD4^+^ and CD8^+^ cells were analyzed by flow cytometry (*D* and *F*). The percentage of p-S6^+^ cells among CD4^+^ and CD8^+^ cells were analyzed by flow cytometry, and is shown as mean ± S.D. (*n* = 3, each group; *E* and *G*). *MFI*, mean fluorescence intensity. *, *p* < 0.05; **, *p* < 0.01. Data are from one experiment (*A–C*) or representative of three animals with similar results (*D–G*).

Some previous studies suggested a connection between the p38-MK2 signaling module and the mechanistic target of rapamycin (mTOR): p38 was required for stress-induced mTOR signaling in arsenite-exposed fibroblasts, hypoxic-reoxygenated cardiomyocytes, and anisomycin-treated macrophages (35–37). Anisomycin-induced mTOR activation has been attributed to the ability of MK2 to phosphorylate and inactivate the mTOR inhibitor TSC2 (37, 38). It remained unexplored, however, whether signaling events consistent with this model occurred in T cells during TCR activation. We found that the loss of MK2 activation in Δαβ T cells was accompanied by a marked reduction in the phosphorylation of TSC2 at serine-1254, which is associated with MK2-dependent inactivation (Fig. 2*A*). In parallel, mTOR pathway activation, as indicated by S6K and S6 phosphorylation, was greatly weakened in Δαβ T cells but persisted in Δα and Δβ T cells, albeit in lower magnitudes than in WT counterparts (Fig. 2, *A* and *B*). TCR-induced S6 phosphorylation was also diminished in CD4^+^ T cells lacking MK2 and the related protein kinase MK3 (Fig. 3, *A–E*), which served overlapping signaling functions (39, 40). Intriguingly, TCR-induced S6 phosphorylation remained intact in CD8^+^ T cells lacking MK2 and MK3 (Fig. 3, *F* and *G*). In summary, p38-dependent activation and regulation of intracellular signaling events in naïve CD4^+^ T cells were abolished by ablation of both p38α and p38β but not either alone.

##### Activation and Th Effector Differentiation of T Cells Lacking p38α and p38β

**FIGURE 4.**
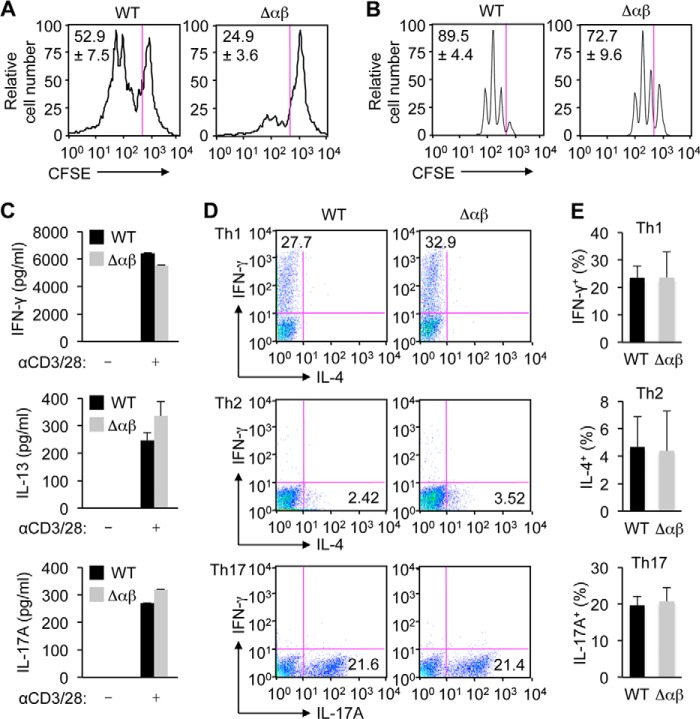
**T cells lacking p38α and p38β remain capable of TCR-induced activation and differentiation into cytokine-producing effector cells *in vitro*.** *A* and *B*, splenic T cells (*A*) and naïve CD4^+^ T cells (*B*) from the indicated mice were labeled with CFSE and stimulated with anti-CD3 and anti-CD28. After 72 h of stimulation, cell proliferation was analyzed by determining the percentage of cells with CFSE dilution among CD4^+^ cells by flow cytometry. The percentages of divided cells are shown as mean ± S.D. (*n* = 3, each group). *C*, naïve CD4^+^ T cells from the indicated mice were left unstimulated or stimulated with anti-CD3 and anti-CD28 (αCD3/28). After 48 h of stimulation, IFN-γ, IL-13, and IL-17A amounts in the culture supernatants were determined by ELISA, and are shown as mean ± S.D. (*n* = 2 for IFN-γ and IL-13, each group; *n* = 3 for IL-17A, each group). *D* and *E*, naïve CD4^+^ T cells from the indicated mice were stimulated with anti-CD3, anti-CD28, and simultaneously treated with varying agents for 5 days to induce Th1 (*top*), Th2 (*middle*), and Th17 (*bottom*) cell differentiation. Effector T cell differentiation was analyzed by determining the percentage of cells with the indicated intracellular cytokines by flow cytometry. Cell percentages are shown as mean ± S.D. (*n* = 3, each group). Data are from one experiment (*A–E*).

We sought to identify functional defects in T cells that resulted from loss of p38 signaling. First, we isolated total (CD3^+^) splenic T cells from WT and αβΔ mice and stimulated them with anti-CD3 and anti-CD28 antibodies. The proliferation rate of TCR-stimulated CD4^+^ T cells was substantially reduced by p38α and p38β deficiency (Fig. 4*A*). These effects could indicate a cell-autonomous role for p38 signaling in TCR-induced proliferation or, alternatively, a contribution of a non-cell-autonomous mechanism that suppressed T cell activation more effectively without the p38 isoforms. Treg cells, contained in the total splenic T cell preparations, might exert the suppressive effect under the latter scenario.

To exclude or minimize the possible influence of Treg cells, we performed subsequent experiments using cell preparations devoid of antigen-experienced T cells including Foxp3^+^ cells. To this end, the CD4^+^CD62L^+^ subset was purified from splenic T cells and subjected to TCR stimulation. Naïve T cells thus prepared from Δαβ mice proliferated at only moderately reduced rates (Fig. 4*B*) compared with the effects seen with total splenic T cells. Naïve CD4^+^ T cells from WT and Δαβ mice produced comparable amounts of IFN-γ, IL-13, and IL-17A upon TCR stimulation (Fig. 4*C*), and differentiated into the effector cell subsets specialized for producing these cytokines (Th1, Th2, and Th17) with similar efficiencies (Fig. 4, *D* and *E*). Therefore, p38 signaling in T cells seemed largely dispensable for TCR-driven activation and differentiation toward Th effector cells.

##### Treg Differentiation of T Cells Lacking p38α and p38β

**FIGURE 5.**
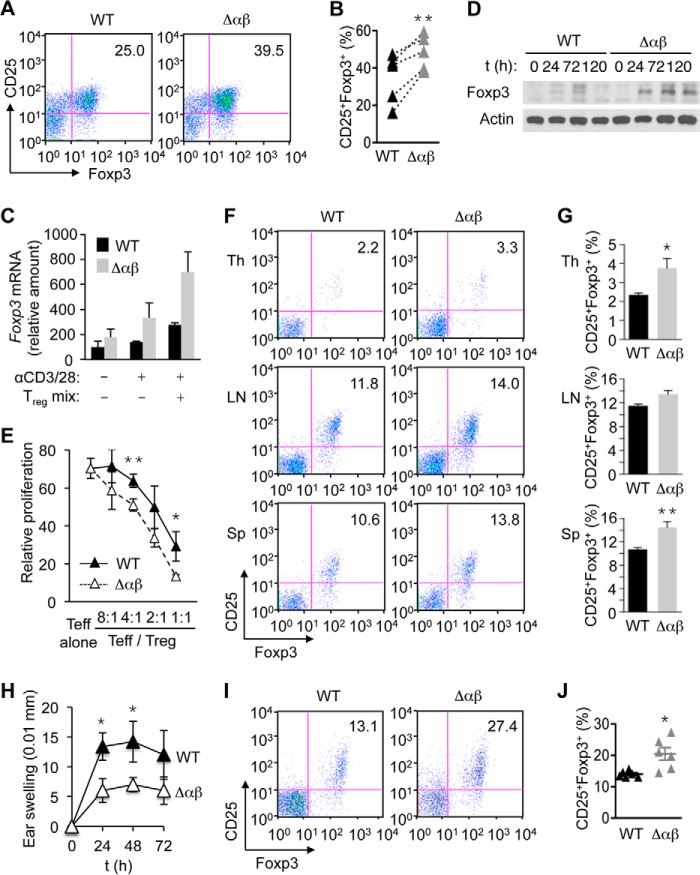
**Deficiency of p38α and p38β in T cells leads to increased Treg cell induction.** *A* and *B*, naïve CD4^+^ T cells from the indicated mice were cultured in the presence of anti-CD3, anti-CD28, IL-2, TGF-β, anti-IFN-γ, and anti-IL-4 for 5 days, and analyzed for CD25 and Foxp3 expression by flow cytometry. Values connected with *dotted line* indicate cell percentages from the same experiment (*B*). **, *p* < 0.01 (the paired Student's *t* test). *C*, naïve CD4^+^ T cells from the indicated mice were left unstimulated, stimulated with anti-CD3 and anti-CD28 (αCD3/28) alone, or stimulated with αCD3/28 in conjunction with treatment with IL-2, TGF-β, anti-IFN-γ, and anti-IL-4 (Treg mix). After 72 h of treatment, total RNA was isolated, and *Foxp3* expression was analyzed by quantitative real-time PCR (*n* = 2). *D*, naïve CD4^+^ T cells from the indicated mice were stimulated with αCD3/28 in conjunction with treatment with the Treg mix. Whole cell lysates were prepared at the indicated time points, and Foxp3 protein was analyzed by immunoblotting. *E*, CFSE-labeled naïve CD4^+^ T cells were treated with anti-CD3 and anti-CD28 in the absence (Teff alone) and presence of Treg cells from WT and αβΔ mice at the indicated ratios (Teff/Treg). After 3 days of stimulation, cell proliferation was analyzed by determining the percentage of cells with CFSE dilution by flow cytometry. Relative proliferation is shown as mean ± S.D. (*n* = 3, each group). *F* and *G*, percentage of CD25^+^Foxp3^+^ cells among CD4^+^ cells in the indicated mouse tissues was determined by flow cytometry (*F*) and shown as mean ± S.E. (*n* = 7, each group; *G*). *, *p* < 0.05; **, *p* < 0.01. *H–J*, indicated mice were sensitized and challenged with the hapten DNFB. Hapten-specific skin swelling was determined at the indicated time points after challenge and shown as mean ± S.D. (*n* = 4, each group; *H*). The percentage of CD25^+^Foxp3^+^ cells among CD4^+^ cells in the draining lymph nodes of the indicated mice was determined by flow cytometry 72 h after hapten challenge (*I*) and shown as mean ± S.E. (*n* = 6, each group; *J*). *, *p* < 0.05. Data are representative of five (*A* and *B*), two (*C* and *D*), or three experiments (*E–G*) with similar results, or from one experiment (*H–J*).

We examined whether p38 signaling played a role in the induction of Treg cells. Upon TCR stimulation in conjunction with exposure to TGF-β, naïve CD4^+^ T cells from Δαβ mice differentiated into Treg cells more efficiently than WT counterparts (Fig. 5, *A* and *B*). Consistently, T cells from Δαβ mice produced greater amounts of Foxp3 mRNA and protein than WT cells during the course of Treg cell induction (Fig. 5, *C* and *D*). TCR-responsive Foxp3 mRNA induction in the absence of a Treg-skewing condition was also higher in naïve CD4^+^ T cells from Δαβ mice (Fig. 5*C*), suggesting that p38 signaling served to limit Foxp3 expression even before the Treg phenotype was established. The suppressive function of Treg cells from Δαβ mice, as determined by the ability to inhibit TCR-induced proliferation of naïve CD4^+^ T cells, was intact, and in fact slightly enhanced compared with that of WT Treg cells (Fig. 5*E*).

We next investigated Treg cell formation *in vivo*. Notably, Δαβ mice in a steady-state condition exhibited a small but consistent increase in Treg cell frequency: CD4^+^ T cells from the thymus and spleen of Δαβ mice contained elevated Foxp3^+^ fractions (Fig. 5, *F* and *G*). To examine Treg cell induction during T cell-mediated immune responses, we subjected WT and Δαβ mice to an experimental condition that elicited contact hypersensitivity reactions. In this model of allergic dermatitis, epicutaneous exposure to a small-molecule antigen or hapten establishes T cell immunity and, upon re-encounter with the hapten, induces T cell-mediated inflammation accompanied by skin rashes and swelling. Δαβ mice displayed greatly attenuated responses to the hapten 2,4-dinitrofluorobenzene (DNFB; Fig. 5*H*) and, simultaneously, a massive expansion of the Treg cell compartment in the skin-draining lymph node (Fig. 5, *I* and *J*).

**FIGURE 6.**
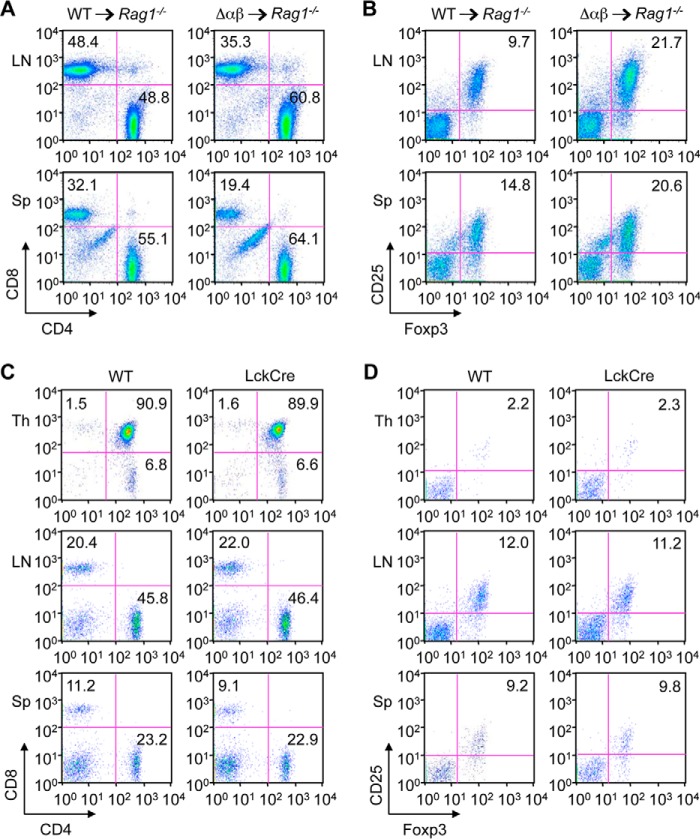
**The T cell-related phenotype of Δαβ mice is not attributable to p38β function in the non-lymphoid compartment or Cre recombinase toxicity.** *A* and *B*, *Rag1*^−/−^ mice were sublethally irradiated and engrafted with bone marrow from WT and Δαβ mice. The percentage of CD4^+^ and CD8^+^ cells among CD3^+^ cells (*A*) and CD25^+^Foxp3^+^ cells among CD4^+^ cells (*B*) in the indicated mouse tissues was determined by flow cytometry 8 weeks after bone marrow transfer. *C* and *D*, percentage of CD4^+^ and CD8^+^ cells among CD3^+^ cells (*C*) and CD25^+^Foxp3^+^ cells among CD4^+^ cells (*D*) in the indicated mouse tissues was determined by flow cytometry. Data are representative of two (*A* and *B*) or three (*C* and *D*) animals per group.

Given the observations described above, p38 signaling was not essential for T cell development and steady-state maintenance. Nevertheless, p38 deficiency in T cells produced perceptible effects in Δαβ mice—altered sizes of CD8^+^ and CD4^+^Foxp3^+^ T cell subpopulations. It was conceivable that these effects resulted from impaired p38 signaling in cell types other than T cells, as Δαβ mice had systemic p38β deficiency. To address this possibility, we examined the lymphoid tissues of RAG1-deficient mice that were sublethally irradiated and engrafted with bone marrow from Δαβ mice. These radiation chimeras, in which deficiency of not only p38α but also p38β was restricted to lymphocytes, exhibited decreased CD8^+^ and increased CD4^+^Foxp3^+^ T cell subsets compared with control chimeras engrafted with WT bone marrow (Fig. 6, *A* and *B*). Hence, the changes in the frequency of T cell subsets in Δαβ mice were likely attributable to the loss of cell-autonomous p38 function. The influence of Cre recombinase-mediated toxicity, if any, was also ruled out based on the normal ranges of CD8^+^ and CD4^+^Foxp3^+^ T cell abundance in *LckCre* mice (Fig. 6, *C* and *D*).

##### Role of p38α/β-MK2/3-mTOR Signaling in Treg Cell Induction

**FIGURE 7.**
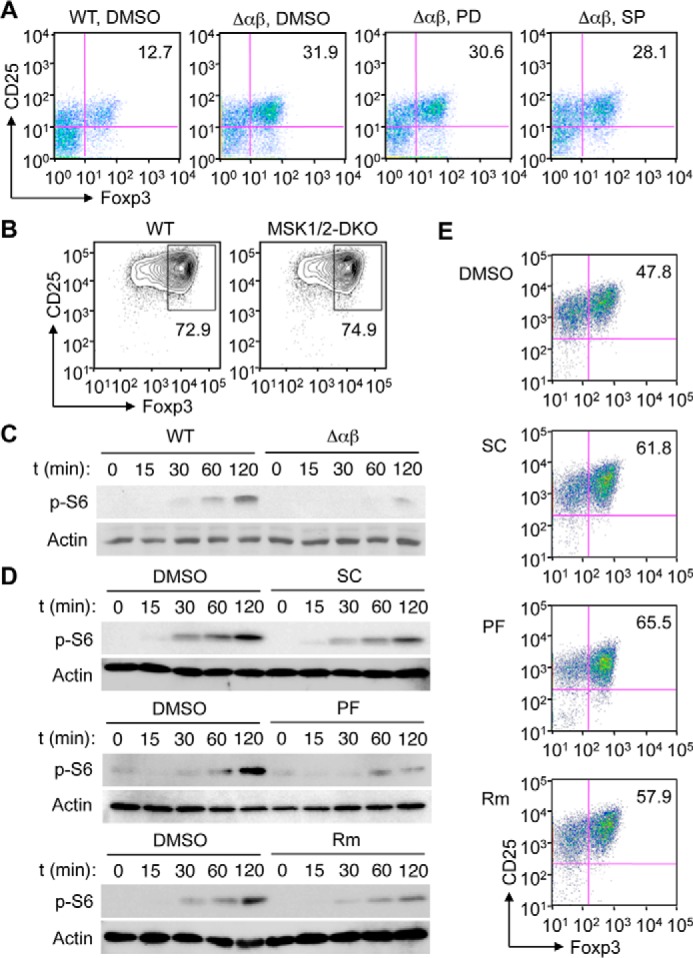
**The p38α/β-MK2/3-mTOR signaling module regulates Treg cell induction.** *A* and *B*, naïve CD4^+^ T cells from the indicated mice were cultured in a Treg-skewing condition for 5 days as in Fig. 5*A*. The cells were treated with vehicle (DMSO), PD98059 (*PD*), and SP600125 (*SP*) throughout the culture period (*A*) or without any of these compounds (*B*). CD25 and Foxp3 expression was analyzed by flow cytometry. *C* and *D*, naïve CD4^+^ T cells from the indicated mice were left unstimulated or stimulated with anti-CD3 and anti-CD28. The cells were left untreated (*C*) or treated with vehicle (DMSO), the p38 inhibitor SC409 (*SC*), the MK2 inhibitor PF3644022 (*PF*), and rapamycin (*Rm*) 1 h before stimulation (*D*). Whole cell lysates were prepared after the indicated durations of stimulation and analyzed by immunoblotting. *p-*, phosphorylated. *E*, naïve CD4^+^ T cells were cultured in a Treg-skewing condition for 5 days as in Fig. 5*A* in the presence of the indicated agents throughout the culture period. CD25 and Foxp3 expression was analyzed by flow cytometry. Data are representative of two (*A* and *E*) or three experiments (*B–D*) with similar results.

Our analysis of Δαβ mice revealed novel effects of loss of p38 signaling in CD4^+^ T cells: increased Foxp3 expression and enhanced induction of fully functional Treg cells. We sought to identify the molecular mechanism whereby p38 signaling modulated Treg cell induction, and explored the possible involvement of the protein kinases whose activation was regulated by or dependent on p38. Excessive activation of ERK and JNK, seen in TCR-stimulated Δαβ T cells (Fig. 2, *A* and *B*), might contribute to enhancing Treg cell induction; we refuted this possibility, however, as pharmacological inhibition of their activation did not significantly reduce the efficiency of Treg cell induction from Δαβ T cells (Fig. 7*A*). Further, deficiency of mitogen- and stress-activated kinase (MSK) 1 and MSK2, which are phosphorylated by and relay signals from ERK and p38 (6), did not profoundly affect Treg cell induction (Fig. 7*B*).

As shown above, S6 protein phosphorylation, an event downstream of mTOR activation, was induced in WT CD4^+^ T cells upon TCR stimulation but substantially diminished in Δαβ and MK2/3-double knock-out (DKO) counterparts (Fig. 2, *A* and *B*; and Fig. 3, *A–E*). We therefore explored MK2/3 and mTOR as possible links for p38-dependent modulation of Treg cell induction. Indeed, pharmacological inhibition and genetic ablation of mTOR have been shown to enhance Foxp3 expression in CD4^+^ T cells, and promote their differentiation into Treg cells (41, 42), paralleling the effect of loss of p38 signaling. To verify the role of p38α/β-MK2/3-mTOR signaling in regulating Treg cell induction, we tested small-molecule compounds acting on distinct points of this protein kinase cascade: SC409, PF3644022, and rapamycin. These compounds, inhibiting p38, MK2, and mTOR, respectively, suppressed TCR-induced S6 phosphorylation as did genetic ablation of p38 (Fig. 7, *C* and *D*). Further, these compounds augmented *in vitro* Treg cell induction to similar extents (Fig. 7*E*).

**FIGURE 8.**
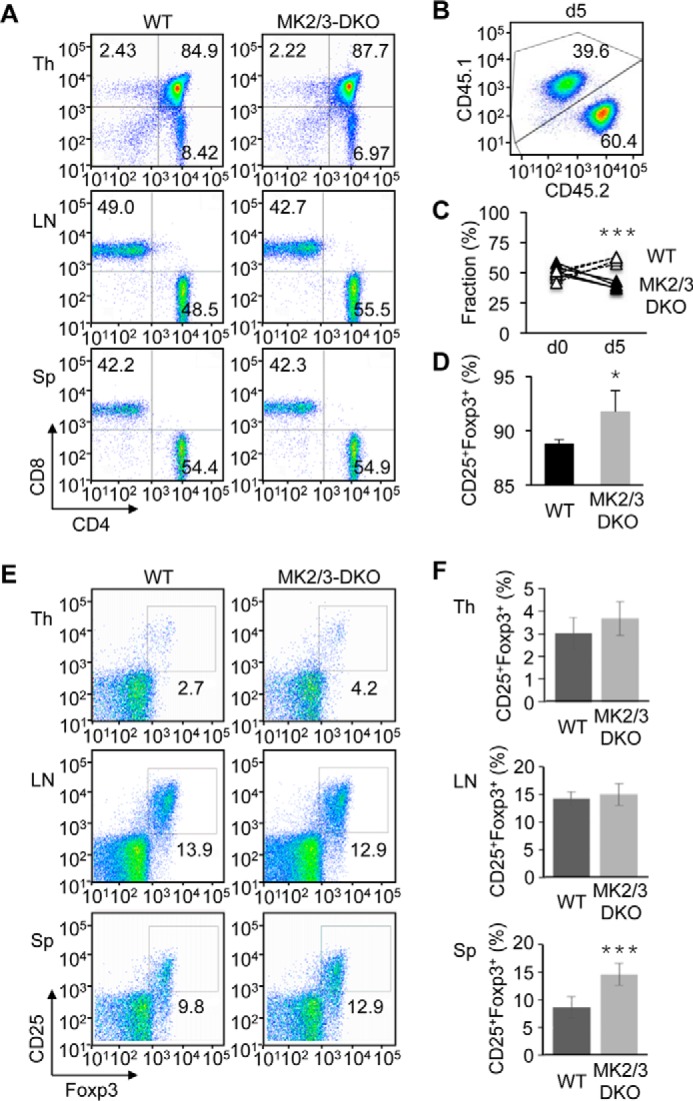
**Genetic ablation of MK2 and MK3 expression leads to enhanced Treg cell induction.** *A*, percentage of CD4^+^ and CD8^+^ cells in the indicated mouse tissues was determined by flow cytometry. *B–D*, equal numbers of naïve CD4^+^ T cells from WT (CD45.1) and MK2/3-DKO (CD45.2) mice were mixed and analyzed before (day 0) and 5 days after culture in a Treg-skewing condition as in Fig. 5*A*. The percentage of CD45.1^+^ and CD45.2^+^ cells (*B* and *C*) and CD25^+^Foxp3^+^ cells among TCRβ^+^CD4^+^ cells (*D*) was determined by flow cytometry. Cell percentages are shown as mean ± S.D. (*n* = 4, each group). *, *p* < 0.05; ***, *p* < 0.001. *E* and *F*, percentage of CD25^+^Foxp3^+^ cells among TCRβ^+^CD4^+^ cells in the indicated mouse tissues was determined by flow cytometry (*E*) and shown as mean ± S.D. (*n* = 7, each group; *F*). ***, *p* < 0.001. Data are representative of two (*A*) or four (*B–D*) experiments with similar results or from one experiment (*E* and *F*).

To obtain genetic evidence for MK2/3-dependent regulation of Treg cell induction, we investigated T cells isolated from MK2/3-DKO mice. Double deficiency of MK2 and MK3 in mice did not perturb the development and maintenance of CD4^+^ and CD8^+^ T cells (Fig. 8*A*). We compared the relative fitness of WT and MK2/3-DKO T cells in Treg cell formation *in vitro* by mixing equal numbers of naïve CD4^+^ T cells from WT CD45.1^+^ mice and MK2/3-DKO CD45.2^+^ mice and subjecting them to a Treg-skewing condition. The contribution of CD45.2^+^ cells to the Treg cell pools obtained at day 5 was greater than that of CD45.1^+^ cells (Fig. 8, *B* and *C*). The efficiency of naïve-to-Treg conversion was also significantly higher with MK2/3-DKO T cells (Fig. 8*D*). In addition, similar to Δαβ mice, MK2/3-DKO mice exhibited a subtle but significant increase in steady-state Treg cell frequency in the spleen (Fig. 8, *E* and *F*). In summary, our findings point to the p38α/β-MK2/3-mTOR cascade as a new actionable target for promoting the induction of immunosuppressive Treg cells.

## Discussion

A role for the p38 signaling pathway in immune responses was suspected early on, and the promise of effectively treating immune-mediated diseases by suppressing this pathway has been driving efforts to understand how this pathway contributes to immunity and inflammation. The findings of this study suggest a role for p38 signaling in several aspects of T cell biology: mice with T cell-restricted ablation of p38α and p38β expression exhibited reduced numbers of T cells and increased frequencies of Treg cells in lymphoid tissues. The decreases in contact hypersensitivity reactions in these mice were likely attributable to either or both of these two effects. From an extensive investigation of protein kinases activated downstream of the TCR, we identified the p38α/β-MK2/3-mTOR cascade as a key signaling module regulating Treg cell induction while ruling out the involvement of ERK, JNK, and MSK. Pharmacological agents interfering with this multilayered protein kinase module may improve the efficiency of generating or expanding Treg cells *ex vivo* for adoptive cell transfer therapy.

TCR and cytokine receptors play key roles in Treg cell development and function, transmitting intracellular signals that are integrated to induce Foxp3 expression in naïve CD4^+^ T cells and stabilize it in Treg-committed cells. Cytokines provide major cues for the skewing of CD4^+^ T cell differentiation, but the strength of TCR signaling also contributes to determining the fate of activated T cells and, in particular, the efficiency of Treg cell formation (43, 44). Signaling by p38 may be pivotal to interpreting the intensity of TCR activation and tuning Treg signature expression accordingly. We have demonstrated that the loss of p38 signaling in T cells is associated with enhanced Treg cell induction. This effect is in accord with the requirement for p38 in TCR-induced mTOR activation. While we note MK2/3-mediated phosphorylation of TSC2 as a potential mechanistic link between p38 and mTOR, it is also possible that MK2/3 may act on a signaling event upstream of TSC2 as the two MKs have been found to affect Toll-like receptor-induced production of phosphatidylinositol 3,4,5-trisphosphate, a phospholipid activator of AKT-mTOR signaling (45). Of note, there were earlier reports of Treg cell induction enhanced by genetic ablation of other protein kinases downstream of TCR, as seen with T cells lacking the TEC family kinase ITK or doubly deficient for the MAPK kinase kinases MEKK2 and MEKK3 (20, 46). Intriguingly, TCR-induced p38 and mTOR activation was impaired in these T cells (20, 46, 47). It remains to be seen whether the p38-MK2/3-mTOR module serves as a nodal point of intracellular signaling in T cells where upstream inputs are integrated into a scalable signal for Foxp3-driven Treg cell induction.

## Experimental Procedures

### 

#### 

##### Animals

Δα (*Mapk14^fl/fl^-LckCre*), Δβ (*Mapk11*^−/−^), and MAPK-activated protein kinase (MK) 2/3-double knock-out (DKO) mice were previously described (16, 40, 48). Δαβ (*Mapk14^fl/fl^-LckCre-Mapk11*^−/−^) mice were generated by crossing Δα and Δβ mice. All mice were on a C57BL/6 background. Contact hypersensitivity to DNFB was induced as described (16). For bone marrow engraftment, 8-week-old *Rag1*^−/−^ mice (The Jackson Laboratory) were sublethally irradiated (450 rads), engrafted with bone marrow cells (4 × 10^6^) from WT and Δαβ mice, and maintained on sulfamethoxazole- and trimethoprim-supplemented drinking water for 8 weeks prior to analysis. All animal experiments were conducted under IACUC-approved protocols.

##### Reagents

DNFB, brefeldin A, and PF3644022 were from Sigma-Aldrich; SC409, PD98059, SP600125, and rapamycin from EMD Millipore; CFSE from Life Technologies; IFN-γ, IL-2, IL-4, IL-6, and TGF-β from Peprotech. Fluorescent-conjugated antibodies against the following markers were used in flow cytometry: CD3 (145–2C11), CD4 (GK1.5 and RM4–5), CD8 (53–6.7), CD25 (PC61.5), Foxp3 (anti-mouse/rat staining kit), IFN-γ (XMG1.2), IL-4 (BVD6–24G2), IL-17A (eBio17B7; all from eBioscience). Antibodies against the following proteins were used in cell stimulation, Fc receptor blocking, and cytokine neutralization: CD3 (145–2C11), CD28 (37.51), IFN-γ (XMG1.2), IL-12 (C17.8), IL-4 (11B11), CD16/CD32 (2.4G2; all from BD Pharmingen); and hamster IgG (MP Biomedicals). Antibodies against the following proteins were used in immunoblotting: ERK (9102), phosphorylated (p-) p38 (9211), p-JNK (9251), p-ERK (9101), p-S6K1 (9205), p-S6 (4858), p-MK2 (3007), p-TSC2 (3616; all from Cell Signaling Technology); p38α (sc-535; Santa Cruz Biotechnology); p38β (33–8700; Life Technologies); p38γ and p38δ (University of Dundee); JNK (554285, BD Pharmingen); Foxp3 (14–5773, eBioscience); and actin (A4700; Sigma-Aldrich).

##### T Cell Isolation and Flow Cytometry

CD3^+^ T cells and naïve CD4^+^ T cells were isolated from the thymus, lymph nodes, and spleen of 6–8-week-old mice using the Pan T cell Isolation Kit and the CD4^+^CD62L^+^ T Cell Isolation Kit, respectively (Miltenyi Biotec). Single-cell suspensions obtained from mice were incubated with Fc receptor-blocking anti-CD16/CD32, stained with fluorescent-conjugated antibodies, and analyzed by flow cytometry using FACSCanto and LSRFortessa (BD Biosciences) and FlowJo software (Tree Star).

##### T Cell Activation in Vitro

Naïve CD4^+^ T cells from the spleen were stimulated with plate-bound anti-CD3 (10 μg/ml) and soluble anti-CD28 (2 μg/ml). Carboxyfluorescein diacetate succinimidyl ester (CFSE)-labeled T cells were stimulated with anti-CD3 and anti-CD28 for 3 days, and analyzed by flow cytometry. To analyze Treg suppressive function, lymph node and splenic CD4^+^CD25^+^ cells were isolated using the Regulatory T Cell Isolation Kit (Miltenyi Biotec). CFSE-labeled naïve CD4^+^ T cells were mixed with CD4^+^CD25^+^ cells, stimulated with anti-CD3 and anti-CD28 for 3 days, and analyzed for proliferation.

##### T Cell Differentiation in Vitro

To induce T cell differentiation into Th effector cells, naïve CD4^+^ T cells from the spleen were incubated with the following agents: Th1, IL-2 (20 ng/ml), IL-12 (10 ng/ml), IFN-γ (10 ng/ml), anti-IL-4 (10 μg/ml); Th2, IL-2 (20 ng/ml), IL-4 (10 ng/ml), anti-IFN-γ (10 μg/ml), and anti-IL-12 (10 μg/ml); and Th17, IL-6 (20 ng/ml), TGF-β (2.5 ng/ml), anti-IFN-γ (2 μg/ml), anti-IL-4 (2 μg/ml), soluble anti-CD28 (1 μg/ml), soluble anti-CD3 (0.25 μg/ml), and plate-bound anti-hamster IgG (0.12 μg/ml). After 5 days of differentiation, cells were treated with phorbol myristate acetate (50 ng/ml) and ionomycin (1 μm) in the presence of brefeldin A (10 μg/ml) for 5 h and analyzed by flow cytometry. For Treg cell induction, naïve CD4^+^ T cells were stimulated with anti-CD3 and anti-CD28 in the presence of IL-2 (20 ng/ml), TGF-β (5 ng/ml), anti-IFN-γ (10 μg/ml), and anti-IL-4 (10 μg/ml) for 5 days. Cells were treated with SC409 (10 μm), PF3644022 (10 μm), and rapamycin (0.1 μm) where indicated.

##### Protein and RNA Analysis

Whole cell lysates were prepared and analyzed by immunoblotting as described (49). Immunoblot signals were quantified by using ImageJ software (National Institutes of Health). Cytokine concentrations in culture supernatants were measured by ELISA. Real-time quantitative PCR was performed using gene-specific primers.

##### Statistical Analysis

Data values are expressed as mean ± S.E. or mean ± S.D. *p* values were obtained with the unpaired, two-tailed Student's *t* test unless otherwise indicated.

## Author Contributions

M. H., K. O., K. G., J. S. C. A., and J. M. P. designed the study and wrote the report. M. H., H. H., T. P., P. R., R. V. S., G. Y. J-A., Y. S., M-K. C., J. S., R. K. C. V., and J. M. P. generated and analyzed p38 and MK KO mice. M. H., T. P., P. R., R. V. S., R. K. C. V., and J. M. P. performed *in vitro* experiments. M. H., P. R., K. O., K. G., J. S. C. A., and J. M. P. interpreted data. All authors reviewed the results and approved the final version of the manuscript.

## Footnotes

3 The abbreviations used are:

TCR: T cell receptor
CFSE: carboxyfluorescein diacetate succinimidyl ester
DKO: double knockout
DNFB: 2,4-dinitrofluorobenzene
MK: MAPK-activated protein kinase
MSK: mitogen- and stress-activated kinase
mTOR: mechanistic target of rapamycin.
